# Modeling the Effect of Temperature on Membrane Response of Light Stimulation in Optogenetically-Targeted Neurons

**DOI:** 10.3389/fncom.2020.00005

**Published:** 2020-02-04

**Authors:** Helton M. Peixoto, Rossana M. S. Cruz, Thiago C. Moulin, Richardson N. Leão

**Affiliations:** ^1^School of Science and Technology (ECT), Federal University of Rio Grande do Norte (UFRN), Natal, Brazil; ^2^Neurodynamics Lab, Brain Institute, Federal University of Rio Grande do Norte, Natal, Brazil; ^3^Developmental Genetics Unit, Neurodynamics Lab, Department of Neuroscience, Uppsala, Sweden; ^4^Electrical Engineering Department, Federal Institute of Paraiba (IFPB), Joao Pessoa, Brazil; ^5^Institute of Medical Biochemistry, Federal University of Rio de Janeiro, Rio de Janeiro, Brazil

**Keywords:** optogenetics, bio-heat, temperature, finite element method, Hodgkin-Huxley model

## Abstract

Optogenetics is revolutionizing Neuroscience, but an often neglected effect of light stimulation of the brain is the generation of heat. In extreme cases, light-generated heat kills neurons, but mild temperature changes alter neuronal function. To date, most *in vivo* experiments rely on light stimulation of neural tissue using fiber-coupled lasers of various wavelengths. Brain tissue is irradiated with high light power that can be deleterious to neuronal function. Furthermore, absorbed light generates heat that can lead to permanent tissue damage and affect neuronal excitability. Thus, light alone can generate effects in neuronal function that are unrelated to the genuine “optogenetic effect.” In this work, we perform a theoretical analysis to investigate the effects of heat transfer in rodent brain tissue for standard optogenetic protocols. More precisely, we first use the Kubelka-Munk model for light propagation in brain tissue to observe the absorption phenomenon. Then, we model the optothermal effect considering the common laser wavelengths (473 and 593 *nm*) used in optogenetic experiments approaching the time/space numerical solution of Pennes' bio-heat equation with the Finite Element Method. Finally, we then modeled channelrhodopsin-2 in a single and spontaneous-firing neuron to explore the effect of heat in light stimulated neurons. We found that, at commonly used light intensities, laser radiation considerably increases the temperature in the surrounding tissue. This effect alters action potential size and shape and causes an increase in spontaneous firing frequency in a neuron model. However, the shortening of activation time constants generated by heat in the single firing neuron model produces action potential failures in response to light stimulation. We also found changes in the power spectrum density and a reduction in the time required for synchronization in an interneuron network model of gamma oscillations. Our findings indicate that light stimulation with intensities used in optogenetic experiments may affect neuronal function not only by direct excitation of light sensitive ion channels and/or pumps but also by generating heat. This approach serves as a guide to design optogenetic experiments that minimize the role of tissue heating in the experimental outcome.

## Introduction

Optogenetics refers to a group of techniques that rely on genetics and optics for the deterministic control or study of (generally excitable) cells from a similar genetic background (Fenno et al., 2011). The radical idea of using light-driven ion channels and pumps from unicellular organisms to modulate neurons was pioneered by Deisseroth, Nagel, and Boyden and has now spread to neuroscience laboratories throughout the world (Knöpfel et al., 2010; Fenno et al., 2011). Limiting factors of the technique include the availability of genetic markers (Lerchner et al., 2014), the invasiveness of the gene delivery and especially difficulties of delivering light throughout large brain volumes (Lerchner et al., 2014). Perhaps for these reasons, optogenetics studies are vastly more common in small animals, especially mice and rats (Aravanis et al., 2007; Madisen et al., 2012).

To date, most *in vivo* experiments rely on light stimulation of neural tissue using fiber-coupled lasers of various wavelengths. Blue and yellow lasers are broadly employed for optogenetic experiments, but due to poor penetration of these light frequencies in the brain, high laser power and/or fibers of high numerical aperture are often used to achieve functional stimulation of deep brain regions (Adamantidis et al., 2014; Adelsberger et al., 2014). Hence, brain tissue is irradiated with high light power that can be deleterious to neuronal function, but surprisingly little attention has been paid on the effects of light stimulation itself in optogenetic experiments. Absorbed light generates heat that can lead to permanent tissue damage. Additionally, neuronal excitability is acutely affected by temperature through the changes in Nernst equilibrium potential and by altering the gating properties of ion channels (Andersen and Moser, 1995; Kim and Connors, 2012). Thus, light alone can generate effects in neuronal function that are unrelated to the genuine ‘optogenetic effect'. In modeling studies, an empirical factor (Q_10_) is used to multiply rate constants to add temperature dependence to the classical Hodgkin and Huxley formalism (Fitzhugh, 1966).

Fiber optics delivered light in biological tissues is partially reflected at the fiber-tissue interface and partially transmitted through the tissue. A previous study (Stujenske et al., 2015) demonstrates that light emitted into the brain through fiber optic delivery is sufficient to increase local temperature and cortical firing rates of single neurons during optogenetics experiments. They also show that *in vivo* temperature recordings validate model predictions of heat induction. They provide an optogenetics MATLAB package for predicting light and heat spread in human brain tissue. On the other hand, the study of Arias-Gil and colleagues (Arias-Gil et al., 2016) uses thermal imaging to directly measure temperature rises at the surface of live mouse brains during laser illumination, with wavelengths and intensities typically used for optogenetics. They use a simple logarithmic model to validate their empirical model by predicting the temperature rise caused by pulsed stimulation paradigms.

The absorbed light is converted to heat, radiated in the form of fluorescence and/or consumed in photobiochemical reactions. The time-dependent heat production in brain tissue can be described by the bio-heat equation (Pennes, 1948), in which changes in tissue temperature can be calculated in time and space. These equations can also account for the buffering of temperature by blood perfusion. Furthermore, laser radiation increases stored energy that results in the diffusion of heat away from the irradiated area in proportion to the temperature gradients generated within the tissue (Welch and Van Gemert, 2011). Therefore, the conclusion drawn from optogenetic experiments may be hindered if the direct heat effect of light stimulation is not accounted for.

In this work, we model the optothermal effect in mice brain tissue produced by visible light laser sources (with a Gaussian profile) in both continuous and pulsed modes (Aravanis et al., 2007; Bernstein et al., 2008) to understand how heat can affect the transfer function of single neurons and how it can alter their response to photocurrents. We first approach the time/space numerical solution of Pennes' bio-heat equation comprising the effects of blood perfusion and metabolism with the finite element method (FEM) (Zimmerman, 2004). We then simulate the effect of varying heat in two single neuron models (Wang and Buzsáki, 1996; Rothman and Manis, 2003) that include a voltage and light-dependent current based on the channelrhodopsin-2 dynamics (Williams et al., 2013) to demonstrate that heat itself can considerably alter neuronal dynamics.

## Methods

### Absorption

Absorption is a process involving the extraction of energy from light by a molecular species. It is important in diagnostic and therapeutic applications in biomedical photonics. The concept of the cross section is used for absorption, where the power absorbed is part of the incident intensity. Therefore, for a given absorber, the absorption cross-section, σ_*a*_, can be defined as (Welch and Van Gemert, 2011; Vo-Dinh, 2014):

(1) $$\begin{array}{r} \sigma_{a}\left(\hat{a}\right)=\frac{P_{a}}{I_{w}}, \end{array}$$

where, $\hat{a}$ is the propagation direction of the plane wave relative to the absorber, *P*_*a*_ is the absorbed power, and *I*_*w*_ is the intensity of the wave. Therefore, a medium with absorbing particles can be characterized by the absorption coefficient, μ_*a*_:

(2) $$\begin{array}{r} \mu_{a}=\rho_{a}\sigma_{a}, \end{array}$$

where, ρ_*a*_ represents the numeric density (*m*^−3^) of the absorbers. Similar equations are found in the literature to explain the scattering phenomenon (Welch and Van Gemert, 2011; Vo-Dinh, 2014).

### Refraction

The relation between the angle of incidence, *θ*_1_, and the angle of refraction, *θ*_2_, for the transmitted light is given by Snell's law (Balanis, 2012; Peatross and Ware, 2015):

(3) $$\begin{array}{r} sin\left(\theta_{2}\right)=\frac{n_{1}}{n_{2}}sin\left(\theta_{1}\right). \end{array}$$

Similarly, the relation between the incident wavelength (medium 1) and the refracted wavelength (medium 2) can be obtained by (Vo-Dinh, 2014):

(4) $$\begin{array}{r} \lambda_{2}=\frac{n_{1}}{n_{2}}\lambda_{1}. \end{array}$$

### Photon Flux

Since light frequency does not depend on the refractive index, the photon energy is always the same as in a vacuum, according to (Welch and Van Gemert, 2011; Vo-Dinh, 2014):

(5) $$\begin{array}{r} E=hf, \end{array}$$

where, *h* = 6.626 · 10^−34^ *J* · *s* is Planck's constant and *f* is the photon frequency (*Hz*).

Photon flux in a laser light beam is defined as the total number of photons crossing a particular section of the light beam, per unit area and per unit time (Svelto and Hanna, 2010). The number of photons emitted per second is given by:

(6) $$\begin{array}{r} N_{p}/s=P\frac{\lambda }{hc}, \end{array}$$

in which, *P* is the laser power. Then, the photon flux, *ϕ*_*p*_, can be obtained as a function of the cross section area (*A, m*^2^) of the light beam as well as the intensity (*I, W*/*m*^2^) of the light beam, according to (Svelto and Hanna, 2010):

(7) $$\begin{array}{r} \phi_{p}=\frac{P}{A}\frac{\lambda }{hc}=I\frac{\lambda }{hc}. \end{array}$$

### Gaussian Laser Beam

Assuming that a laser beam in the *z* direction attenuates exponentially with the distance *d* in the tissue (Welch and Van Gemert, 2011), the irradiance can be defined as the radiant energy flux incident on the point of the surface, divided by the area of the surface. Many laser sources emit beams that approximate a Gaussian profile, in which case the propagation mode of the beam is the fundamental transverse electromagnetic mode (*TEM*_00_) (Balanis, 2012; Sadiku, 2014).

Gaussian functions can assume multidimensional forms by composing the exponential function with a concave quadratic function (Weisstein, 2015). A particular example of a two-dimensional Gaussian function, in the *x* − *y* plane, is:

(8) $$\begin{array}{r} f\left(x,y\right)=A\text{ exp}\left[-\left(\frac{{\left(x-x_{0}\right)}^{2}}{2\sigma_{x}^{2}}+\frac{{\left(y-y_{0}\right)}^{2}}{2\sigma_{y}^{2}}\right)\right]. \end{array}$$

Considering a bell curve shape for the Gaussian function, the parameter *A* is the maximum amplitude of the curve, *x*_0_ and *y*_0_ are the center position of the curve in *x* and *y* axis, and σ_*x*_ and σ_*y*_ are the *x* and *y* spreads or standard deviations of the Gaussian curve.

### Light Propagation in Brain Tissue

*In vitro* and *in vivo* optogenetic experiments commonly use a relatively simple setup that consists of laser sources coupled to optical fibers to deliver light to a region of interest (ROI) in the tissue, in an accurate and efficient manner. *In vivo* experiments in deep regions of the brain, for example, also require a stereotactic surgery to position the tip of the optical fiber in the ROI into the brain (Zhang et al., 2015). Depending on the distance from the fiber tip and the optical properties of the surrounding tissue, the emitted light can propagate with uneven intensity.

The transmittance, *T*, is the relationship between the light intensity measured in the tissue at a distance *d*, and the light intensity measured without tissue, $\frac{I\left(d\right)}{I\left(d=0\right)}$, considering both scattering and absorption effects, and is given by (Vo-Dinh, 2014):

(9) $$\begin{array}{r} T=\frac{b}{a\text{ sinh}\left(bd\mu_{s}\right)+b\text{ cosh}\left(bd\mu_{s}\right)}, \end{array}$$

in which, μ_*s*_ is the scattering coefficient and can be given in *mm*^−1^ (Aravanis et al., 2007; Bernstein et al., 2008), *d* is the distance in the brain tissue (*mm*), and *a* and *b* are given by (Vo-Dinh, 2014):

(10) $$\begin{array}{r} a=1+\frac{\mu_{a}}{\mu_{s}}, \end{array}$$

(11) $$\begin{array}{r} b=\sqrt{a^{2}-1.} \end{array}$$

here, μ_*a*_ can also be given in *mm*^−1^ (Aravanis et al., 2007; Bernstein et al., 2008).

The light intensity can be estimated by the product between the transmittance *T* and the geometric loss *g*_*loss*_ due to light spreading in the tissue. The geometric loss is obtained by the decrease in light intensity due to the conical shape observed from the fiber tip (*d* = 0) to a certain distance *d* in the tissue. The divergence angle, *θ*_*div*_, for a multimode fiber is given by (Aravanis et al., 2007):

(12) $$\begin{array}{r} \theta_{div}={\text{sin}}^{-1}\left(\frac{NA_{fib}}{n_{t}}\right), \end{array}$$

where, *n*_*t*_ is the refractive index of the tissue and *NA*_*fib*_ is the numerical aperture of the optical fiber. Considering the conservation of energy, we can calculate the geometric loss, *g*_*loss*_, to a given distance, *d*, in the tissue as (Aravanis et al., 2007):

(13) $$\begin{array}{r} g_{loss}=\frac{\rho^{2}}{{\left(d+\rho \right)}^{2}}, \end{array}$$

with,

(14) $$\begin{array}{r} \rho =r\sqrt{{\left(\frac{n_{t}}{NA_{fib}}\right)}^{2}-1,} \end{array}$$

in which, *r* is the fiber core radius. In this way, the expression for the normalized light intensity, *I_N_* (*mW/mm*^2^), considering scattering, absorption and geometric loss is given by:

(15) $$\begin{array}{r} I_{N}=\frac{I\left(d\right)}{I\left(d=0\right)}=g_{loss}\cdot T. \end{array}$$

We can consider *I*(*d* = 0) as the light intensity at the fiber tip that can be obtained in *mW*/*mm*^2^ simply by:

(16) $$\begin{array}{r} I\left(d=0\right)=\frac{P}{A\eta }, \end{array}$$

where, *P* is the power emitted by the light source (*mW*), *A* = π*r*^2^ is the area of the optical fiber (*mm*^2^), and η is the coupling efficiency between the optical fiber and the light source (dimensionless). We chose η = 1 for all the scattering and absorption simulations.

Finally, the light intensity (*mW*/*mm*^2^) at a region of interest in the tissue, assuming a distance *d* (*mm*) from the fiber tip, is given by:

(17) $$\begin{array}{r} I\left(d\right)=I\left(d=0\right)\cdot I_{N}. \end{array}$$

We used MATLAB commercial software to simulate scattering and absorption characteristics in mice brain tissue. Table 1 shows the parameters and respective values used for these simulations.

**Table 1:** Parameters used in scattering and absorption simulations.

**Parameters**	**Values**	**References**
Fiber core radius (*r*)	0.2 *mm*	dat, 2015
Fiber numerical aperture (*NA*)	0.48	dat, 2015
Fiber core refractive index (*n*_1_)	Blue: 1.4644 Yellow: 1.4587	dat, 2015
Scattering coefficient (μ_*s*_)	Blue: 10.0 *mm*^−1^ Yellow: 9.0 *mm*^−1^	Bernstein et al., 2008
Absorption coefficient (μ_*a*_)	Blue: 0.070 *mm*^−1^ Yellow: 0.027 *mm*^−1^	Bernstein et al., 2008
Laser input power (*P*)	20 *mW*	
Laser coupling fraction (η)	1 or 100%	

### Heat Transfer in Mice Brain Tissue

Heat transfer is a known physical problem already modeled in many areas of knowledge (Ahmed et al., 2019; Taheripour et al., 2019). For biology, heat is inevitable when light propagates and is absorbed by biological tissues.

The traditional bio-heat equation describes the change in tissue temperature over time that can be expressed at a distance *d* in the tissue. Furthermore, blood perfusion occurs in living tissues, and the passage of blood modifies the heat transfer in tissues. Pennes (1948) has established a simplified bio-heat transfer model to describe heat transfer in tissue by considering the effects of blood perfusion, ω_*b*_, and metabolism, *H*_*m*_ (Elwassif et al., 2006; Vo-Dinh, 2014):

(18) $$\begin{array}{r} \rho C_{p}\frac{\partial T}{\partial t}=\nabla \left(k\nabla T\right)-\rho_{b}\omega_{b}C_{b}\left(T-T_{b}\right)+H_{s}+H_{m}, \end{array}$$

where, *ρ* is the tissue density (*kg*/*m*^3^), *C*_*p*_ is the specific heat of the tissue (*J*/*kg*°*C*), *k* is the thermal conductivity of the tissue (*W*/*m*°*C*), *ρ*_*b*_ is the blood density (*kg*/*m*^3^), ω_*b*_ is the blood perfusion (1/*s*), *C*_*b*_ is the specific heat of the blood (*J*/*kg*°*C*), *T* is the temperature of the tissue (°*C*), *T*_*b*_ is the blood temperature (°*C*), *H*_*s*_ is the heat source due to photon absorption (*W*/*m*^3^), and *H*_*m*_ is the term that represents heat generated by metabolism (*W*/*m*^3^). Equation (18) is almost linear for small temperature changes, therefore, it is expected that temperature rises are approximately proportional to the energy input (that is, duty cycle).

The interaction between metabolic heat generation and blood perfusion was investigated, and it was proved that the temperature increases during Deep Brain Stimulation (DBS). Other environmental interactions that can affect the stored energy include radiation and convection from the sample surface, the loss of vapor phase water from the sample, and convection with blood that is perfused through the vascular network from arterial and venous sources. This network has a very specific geometry that is unique to a tissue or organ and can affect significantly the capability to exchange heat with the tissue in which it is embedded (Welch and Van Gemert, 2011).

Additionally, thermal boundary interactions occur over the surface area with the environment and are often characterized as convective and irradiative processes. Laser irradiation process increases the stored energy from its initial state and, as a result, it diffuses the heat away from the irradiated area in proportion to the temperature gradients developed in the tissue. A quantitative characterization of the formation of these gradients and the heat flow that they drive are the focus of heat transfer analysis (Welch and Van Gemert, 2011).

In the case of convective boundary conditions, heat transfer occurs when a solid substrate is in contact with a fluid at a different temperature (Welch and Van Gemert, 2011). The magnitude of the heat exchange can be calculated according to Newton's law of cooling, that describes the convective flow, *H*_*conv*_ (*W*/*m*^2^), at the surface in terms of the convective heat transfer coefficient, *h* (*W*/*m*^2°^C) and the temperatures of the sample, *T*, and the external environment, *T*_*ext*_, in °C:

(19) $$\begin{array}{r} H_{conv}=h\left(T-T_{ext}\right). \end{array}$$

We consider the geometry and shape of the boundary layer region of the fluid in which convection occurs, to calculate the free convective flow. Convective effects are hard to estimate once different process characteristics must be considered depending on the convective transport problem. Typical values of *h* for free convection in liquids are in the range of 20–1,000 (*W*/*m*^2°^*C*) (Welch and Van Gemert, 2011). It is important to choose small values of *h*, such as 25 *W*/*m*^2°^*C*, so that the temperature variations between the environment and the sample are properly evidenced.

Heating generated within the biological material is governed by the following expression (Elwassif et al., 2006):

(20) $$\begin{array}{r} H(x,y,z)=P(1-R)\frac{\mu_{a}}{\pi \sigma_{x}\sigma_{y}}\text{exp}[-\left(\frac{{\left(x-x_{0}\right)}^{2}}{2\sigma_{x}^{2}}\right)\\ +\frac{{\left(y-y_{0}\right)}^{2}}{2\sigma_{y}^{2}}]\text{exp}(-\mu_{a}z), \end{array}$$

in which, the first exponential function represents the two-dimensional Gaussian distribution in *x*−*y* plane, in accordance to Equation (8). The second exponential function represents the exponential decay due to absorption (Yang and Miklavcic, 2005).

Some considerations in using Equation (20) are: the reflection (*R*) and absorption coefficients are assumed to be constant; the sample is assumed to have a planar surface aligned with the *xy*-plane of the global coordinate system and whose top matches *z* = 0 (distance at the fiber tip); the center of the beam can be easily shifted by changing *x*_0_ and *y*_0_; the beam width can be easily controlled by the standard deviation parameters σ_*x*_ and σ_*y*_. We assumed *R* = 0 and σ_*x*_ = σ_*y*_ = 0.5 for the analysis of heat transfer performed in this work.

Heat transfer simulations were accomplished using the computational modeling software, COMSOL Multiphysics 4.4, that allows numerical solutions for partial differential equations based on the Finite Element Method (FEM) (Zimmerman, 2004). Laser heating was simulated considering two stationary conditions: continuous mode and pulsed mode. We used biological material with mice brain tissue characteristics (gray matter). The material properties were assumed to be constant and are shown in Table 2.

**Table 2:** Parameters and material properties used in heat transfer simulations.

**Parameters**	**Values**	**References**
Refractive index of the tissue (*n*_*t*_)	1.36 (gray matter)	Vo-Dinh, 2014
Specific heat of the tissue (*C*_*p*_)	3650 *J*/*kg*°*C*	Elwassif et al., 2006
Density of the tissue (*ρ*)	1040 *kg*/*m*^3^	Elwassif et al., 2006
Thermal conductivity of the tissue (*k*)	0.527 *W*/*m*°C	Elwassif et al., 2006
Metabolic heat (*H*_*m*_)	13698 *W*/*m*^3^	Elwassif et al., 2006
Blood density (*ρ*_*b*_)	1057 *kg*/*m*^3^	Elwassif et al., 2006
Blood perfusion (ω_*b*_)	0.012 1/*s*	Elwassif et al., 2006
Specific heat of the blood (*C*_*b*_)	3600 *J*/*kg*°C	Elwassif et al., 2006
Temperature of the tissue (*T*)	37°C	Elwassif et al., 2006
Blood temperature (*T*_*b*_)	36.7°C	Elwassif et al., 2006
Heat transfer coefficient (*h*)	25 *W*/*m*^2^°C	Welch and Van Gemert, 2011
Standard deviations in *x* and *y* axis (σ_*x*_, σ_*y*_)	0.5	
Reflection coefficient (*R*)	0	

### Channelrhodopsin-2 and Neuron Models

We first modeled the effect of temperature alone in a pyramidal cell model and in a network of basket cells known to generate gamma oscillations. We have implemented a single compartment CA1 neuron model described by Migliore (Migliore, 1996). He has implemented a multicompartment model in his original work, but here we only employ the soma with an inactivating sodium conductance (max. 30 *nS*), a delayed rectifier *K*^+^ conductance (max. 10 *nS*), conductance from an *M* current (max. 0.6 *nS*) and from an *H* current (max. 0.3 *nS*). Kinetics for all currents were download from ModelDB (https://senselab.med.yale.edu/modeldb/, Accession:2937).

In addition, we have used the same *Q*_10_ values for all voltage-gated currents as the original publication (Wang and Buzsáki, 1996). Temperature values from the heat transfer simulation were fed to the neuron model by a “look up time/temperature table” where each rounded ms value corresponded to a single temperature value. Simulations were run for 90 s (30 s for stabilization with constant temperature and 60 s with variable temperature). The model was solved in MATLAB using the built-in solver “ode23”. The interneuron network gamma model was simulated using Neuron with no changing in parameters from the model available from ModelDB (Accession:26997) exception by setting the temperature to 37 or 39°C. These simulations were run for 500 ms with a constant temperature. Note that the original study of Wang and Buzsáki did not account for temperature; however, the uploaded model in ModelDB includes *Q*_10_ for kinetic variables (Wang and Buzsáki, 1996).

Power spectrum density analysis and cross-correlation of action potentials were calculated from spike trains transformed in a series of 0 s (no spike) and 1 s (spike) with 0.1 *ms*-precision (Hilscher et al., 2013). Power spectral density analysis of binary spike series was performed using Welch's method (pwelch command in MATLAB). Cross-correlograms (CCGs) were calculated as described previously (Hilscher et al., 2013) and then smoothed by a moving average filter with a span of 10 ms (Hilscher et al., 2013). Cross-correlations over a lag range of ±0.1 s. Synchrony index (SI) is defined as the maximum value of the CCG.

We have implemented the channelrhodopsin-2 empirical model (Williams et al., 2013) in two single neuron models to test the interaction of temperature and optocurrents: a single basket cell from Wang and Buzsaki network model (Wang and Buzsáki, 1996) and an anteroventral cochlear nucleus bushy cell model (Rothman and Manis, 2003). The equations and parameters from the neuron models can be found in the original publications (Wang and Buzsáki, 1996; Rothman and Manis, 2003) and equations and parameters for channelrhodopsin optocurrents are found in (Williams et al., 2013). All models were implemented in MATLAB (Mathworks), and the codes can be downloaded from https://github.com/cineguerrilha/Neurodynamics/tree/master/Cell_Models.

## Results

**Figure 1:**
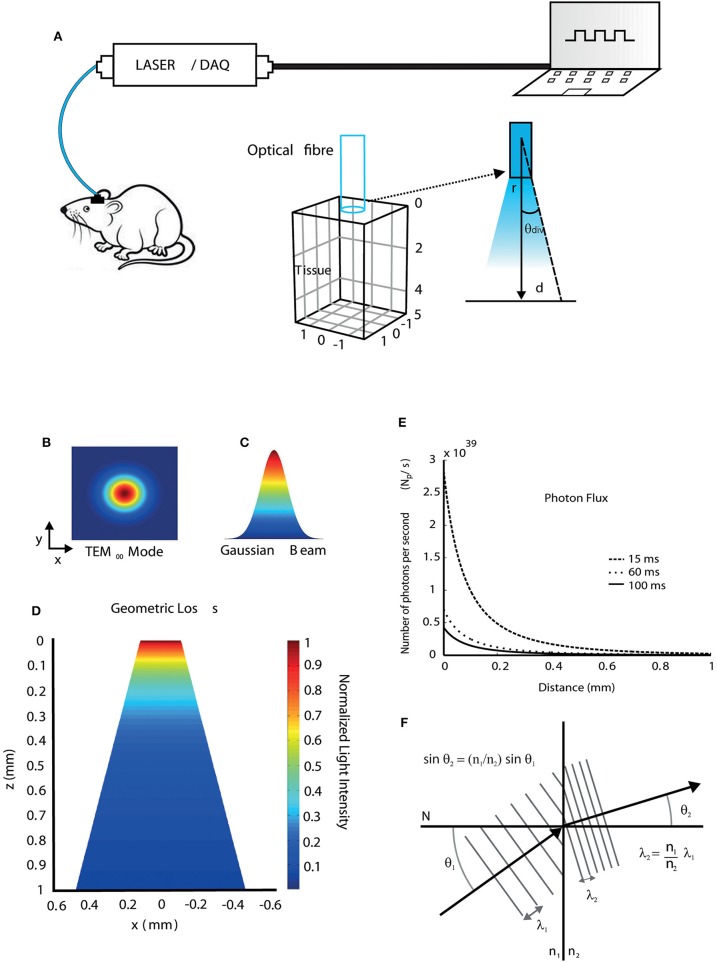
Light propagation properties when interacting with brain tissue. **(A)** Diagram showing a typical optic stimulation setup used in freely moving animals. The setup consists of a computer, a data acquisition (DAQ) board, and a laser source coupled to a fiber transmitting light to a target region into the mouse brain at a divergence angle (*θ*_*div*_) calculated using Equation (12). **(B)** Transversal electromagnetic fundamental propagation mode (*TEM*_00_) of the laser source. **(C)** Gaussian beam shape. **(D)** 2D view of the geometric loss due to light spreading in the tissue (conical shape) at a certain distance from the fiber tip. **(E)** The flux of irradiated photons as a function of distance during 15, 60, and 100 *ms* light pulses considering a region of unit area. **(F)** Wavelength shift during light propagation through different media.

In this work, we first simulated the light propagation and absorption in the brain of mice in a typical optogenetic setup. Figure 1A shows a diode pumped solid state - DPSS laser source coupled to a multimode optical fiber that transmits light directly to the region where the brain implant was performed (Zhang et al., 2015).

Subsequently, we simulated the effect of heat in single neurons and networks. We have also examined the additive effect of heat and light in simulations that included a channelrhodopsin-2 model (Williams et al., 2013). The bio-heat transfer was solved numerically using Pennes' equation with the finite element method and temporal changes in temperature at a given point in space were applied to a single compartment neuron model (with Hodgkin and Huxley formalism).

We first simulated beam geometry and light spreading. A DPSS laser emits a Gaussian beam that the propagation mode is the fundamental transversal electromagnetic (*TEM*_00_) (Figures 1B,C and Equation 8). Figure 1D shows the normalized geometric loss due to light spreading in *z-x* plane within the tissue as a function of the distance from the fiber tip in *z* direction. The divergence angle is determined by the optical fiber numerical aperture, according to Equation (12). After light power at a given point is calculated, photon flux (number of irradiated photons per unit time and per unit area) at that point can be obtained by Equation (7). Photon flux can then be correlated to photocurrents in channelrhodopsin models (Foutz et al., 2012). Photon flux simulations are shown in Figure 1E, in which, a 20 *mW*, 473 nm laser is pulsed with durations of 15, 60, and 100 ms. The different pulse durations were chosen to illustrate that the pulse width changes alter the amount of photons passing through a surface. Light speed is altered during propagation because of the difference of refractive indices and their dependence with wavelength. Consequently, the wavelength can change during propagation and this effect is not only observed in the interface between fiber and tissue, but also within the tissue, due to its anisotropic refractive indexes between different brain regions. The wavelength change between two different media, which is calculated using Snell's law (Equation 3), is illustrated in Figure 1F. Assuming that light propagates from an optical fiber (medium 1) to the tissue (medium 2), where N is a perpendicular line to the surface of separation between the two media, and considering *n*_1b_ = 1.4644 as the refractive index of the fiber core at 473 nm, *n*_1y_ = 1.4587 the refractive index of the fiber core at 593 nm, and *n*_2_ = 1.36 the refractive index of the tissue (mouse brain, gray matter), the wavelength shifts for blue (473 nm) and yellow (593 nm) lights due to refraction are 36 nm and 43 nm, respectively, according to Equation (4). Yet small, wavelength shifts have to be considered specially in modeling studies as there is an obvious relationship between wavelength and light absorption in both light-sensitive ion channels and fluorescent proteins (Zhang et al., 2015), even if the photon energy remains the same, once small changes in the wavelength affect the response of the light-sensitive ion channels and fluorescent proteins.

**Figure 2:**
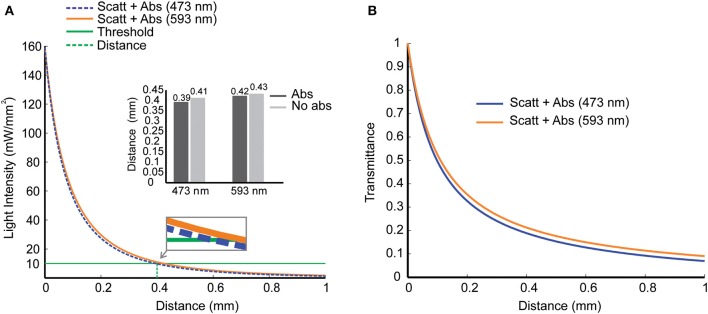
Scattering and absorption effects as light propagate into mouse brain tissue. **(A)** Light intensity vs. penetration distance for 473 *nm* (blue) and 593 *nm* (yellow) wavelengths. At a distance *d* = 0.4 *mm* from the fiber tip (dashed green line), a reference value for light intensity of 10 mW/mm^2^ (solid green line) was chosen for blue and yellow light with (solid lines) and without (dashed lines) absorption. Inset. Distance in which light decays to 10 mW/mm^2^ in simulations with and without absorption. **(B)** Transmittance vs. penetration distance for blue and yellow lights, including scattering and absorption effects.

We then used the Kubelka-Munk model to calculate light intensity vs. distance considering absorption (Mobley and Vo-Dinh, 2003). Light absorption by the tissue has no direct relation to the production of photocurrents by channelrhodopsin; however, absorption produces heat, a side effect of light stimulation (Shapiro et al., 2012). Light absorption also changes (although slightly) the relation between light intensity and tissue depth (Figure 2A). Assuming a threshold of 10 *mW*/*mm*^2^ (green line), which is a sound intensity value when stimulating a large group of stimulated cells (Bernstein et al., 2008), the depth for channelrhodopsin-2 activation is 0.39 *mm* (473 *nm*) and for halorhodopsin activation is 0.42 *mm* (593 *nm*). Figure 2B shows the transmittance (Equation 9) as a function of distance *d*, considering both scattering and absorption effects. These simulations indicate that only cells and neurites at the vicinity of the fiber are affected by light stimulation and are in agreement with a previous study (Stujenske et al., 2015).

We next computed the production of heat in the tissue caused by light absorption using FEM. For heat transmission simulations, we used a rectangular prism of dimensions equal to 3.5 × 3.5 × 5 (*mm*^3^) representing a mouse brain tissue. Optogenetic experiments often use specific stimulation protocols with yellow light to activate halorhodopsin and blue light to activate channelrhodopsin (Cardin et al., 2009; Mikulovic et al., 2016). We, therefore, simulated the interaction between the mouse brain and the yellow light radiation (593 nm wavelength), with the laser source operating in continuous mode, while the blue light radiation (473 nm wavelength) laser source operating in pulsed mode.

**Figure 3:**
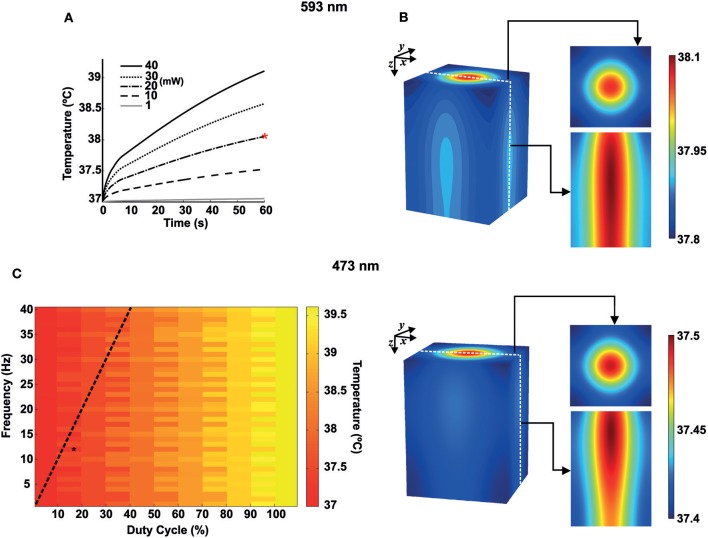
Heat transfer simulations for blue and yellow light in mouse brain tissue. **(A)** Temperature variations for 593 *nm* wavelength as a function of time for 1, 10, 20, 30, and 40 *mW* of continuous radiation. The red asterisk indicates continuous yellow light radiation for 20 *mW* and 60 s. **(B)** Temperature distribution in space for 593 *nm* and 473 *nm*. Right. Top. 2D Gaussian beam (*x-y*) for the top view and with *z* → 0. Bottom 2D slice view (*z-x*) of the temperature distribution. **(C)** Heat map for the temperature distribution (473 *nm*) as a function of frequency (1–40 *Hz*, bin size of 1 *Hz*) and duty cycle (1–100%, bin size of 10%) at 60 *s* of light radiation (10 μ*m* from the fiber tip). The black asterisk indicates pulsed blue light radiation, 12 *Hz* and 18% of duty cycle. The dashed black line shows a pulse width of 10 *ms*.

Temperature changes at a distance *d* = 10 μ*m* from the fiber tip caused by continuous light radiation (593 *nm*) as a function of time are shown in Figure 3A. We simulated heat transfer due to continuous yellow light for different values of power emitted by the laser source: 1, 10, 20, 30, and 40 *mW*. According to Figure 3A, during the first 5 s, the rate of temperature variation is higher. After that, the temperature continues to increase more slowly moving toward the steady state condition. For light power up to 10 *mW*, temperature increases about 0.5°C. For 20, 30, and 40 *mW*, the increase in temperature after 1 min of radiation is between 1 and 2°C. Figure 3B shows a temperature distribution in 3D view, 2D top view (*x-y*), and 2D slice center view (*z-x*, constant *y*), for continuous yellow light radiation (20 *mW* and 60 s, indicated by the red asterisk shown in Figure 3A and pulsed blue light radiation (473 nm), 12 Hz and 18% of duty cycle-percentage of a period in which the light is turned on (black asterisk indicated in Figure 3C). We have also computed temperature changes for 20 *mW* blue light, at 60 s and 10 μ*m* from the fiber tip, for frequencies varying from 1 to 40 *Hz* with duty cycles varying from 1% to 100% (Figure 3C). These results show that lower duty cycles minimize temperature changes by light stimulation.

**Figure 4:**
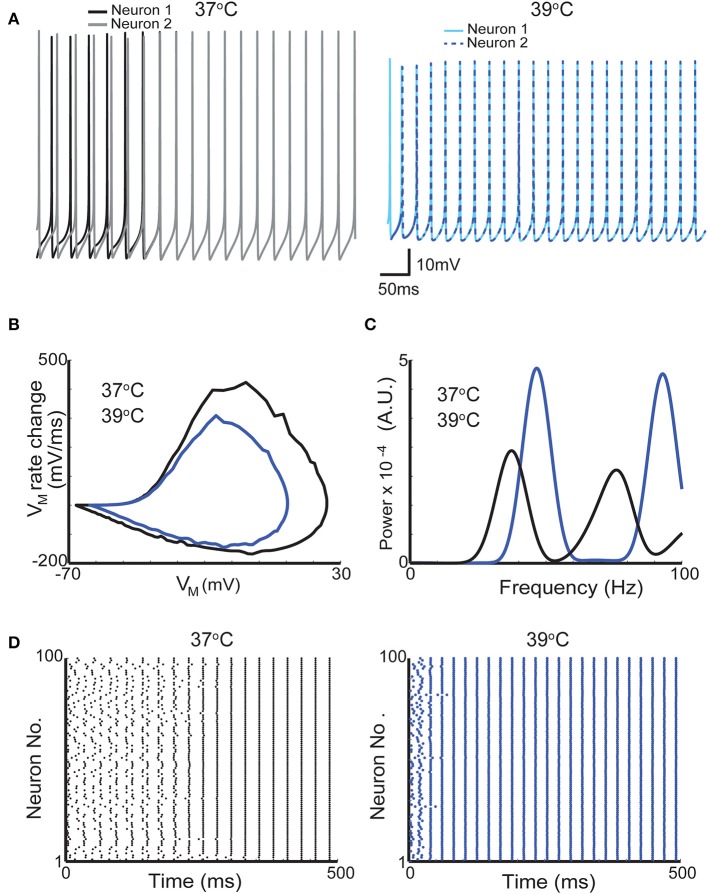
A 2°C raise in temperature increases the firing frequency of neurons in a network model of gamma oscillations. **(A)** Membrane potential of two neurons from a network of 100-interneuron network when simulation was executed with temperatures of 37°C (gray and black traces left) and 39°C (blue and dashed dark blue right). **(B)** Phase plots from one action potential of one interneuron at 37°C and at 39°C (black and dark blue traces, respectively). **(C)** Mean firing power spectrum density (see section Methods) of the 100 interneurons in the network at 37°C and at 39°C (black and dark blue traces, respectively). **(D)** Scatter plots showing the action potential firing of the gamma network at 37°C (left) and at 39°C (right).

Currents produced by voltage-gated ion channels are directly influenced by temperature. It is known for decades that channel opening and closing are generally faster in higher temperatures and conductance/voltage relationship and ion reversal potential are also be affected by temperature (Fitzhugh, 1966). To illustrate the effect of temperature in firing, we used a basket cell model (Wang and Buzsáki, 1996). For these simulations, we used two temperatures (37°C and 39°C the latter can be quickly produced by a pulsed laser at 40 *Hz* and 90% duty cycle and at 10 μm distance from the center of the fiber tip Figure 4). In the model implemented here, action potentials become smaller and briefer (Figures 4A,B). Spontaneous firing frequency of the neuron used in this simulation also increases (Figure 4C). Optogenetics has been used to study the mechanisms behind neuronal synchrony and brain rhythm generation (Cardin et al., 2009). Hence, we further investigated the effect of heat generated by light stimulation itself (rather than photocurrents in channelrhodopsin-expressing neurons) in a network model comprised solely by basket cells that synchronize in gamma frequency (Wang and Buzsáki, 1996). The model is composed of 100 interconnected fast spiking interneurons (same as in Figure 4) (Wang and Buzsáki, 1996). In the Wang and Buzsákis model (Wang and Buzsáki, 1996), neurons in the network take around 200–300 ms to fire in gamma frequency from a relatively asynchronous onset (Figures 4A,D). If the temperature is raised by 2°C the network is synchronized in less than 50 ms (Figures 4A,D) from the onset of simulation. Firing frequency of the interneurons in the network also increased by raising the temperature in 2°C (Figure 4C). This changing in frequency caused a shift in the peak of ‘gamma oscillation' in the power spectrum (Figure 4C). Hence, heat itself can theoretically facilitate the generation of oscillations and/or alter their frequency.

**Figure 5:**
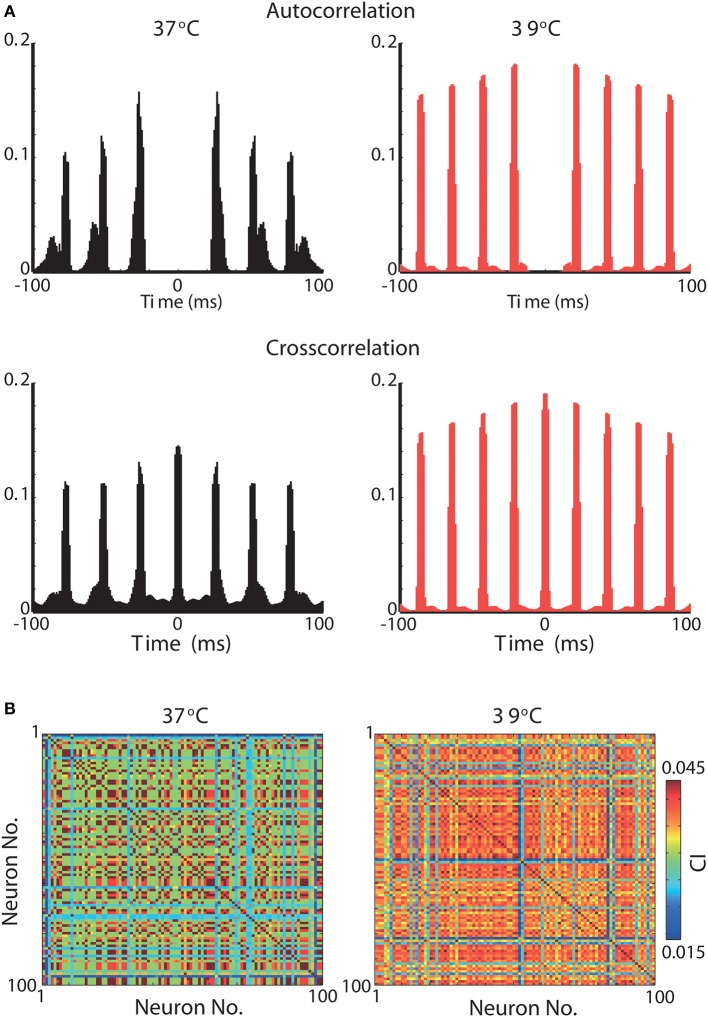
Synchrony is greatly increased in a gamma oscillation network model by a 2°C raise in temperature. **(A)** Top, Normalized autocorrelograms of all 100 neurons in the network at 37°C (left) and at 39°C (right). Bottom, Normalized crosscorrelograms of all 100 neurons crosscorrelated with all 100 neurons in the network at 37°C (left) and at 39°C (right). **(B)** Peak normalized correlation index between all 100 neurons when simulations were performed at temperatures of 37°C (left) and 39°C (right).

We further assess the effect of raising the temperature in neuronal synchronization using previously described synchrony metrics (Leao et al., 2005; Hilscher et al., 2013). Autocorrelation histograms of all 100 neurons in the model are shown in Figure 5A for 37°C and at 39°C. Heating the network model caused neurons to fire at greater rhythmicity (Figure 5A). In addition, cross-correlogram also showed greater synchrony when simulations were executed at 39°C (compared to 37°C). This increase in synchrony is reflected by a significant rise in the synchronization coefficient (Figure 5B). The mean synchronization index (SI) for all possible neuron pair combinations (9,900 pairs) was equal to 0.16 for 37°C and 0.22 for 39°C. These results show that heating can, not exclusively, change the frequency of brain oscillations but also alter the coordination and synchrony of neuronal firing.

**Figure 6:**
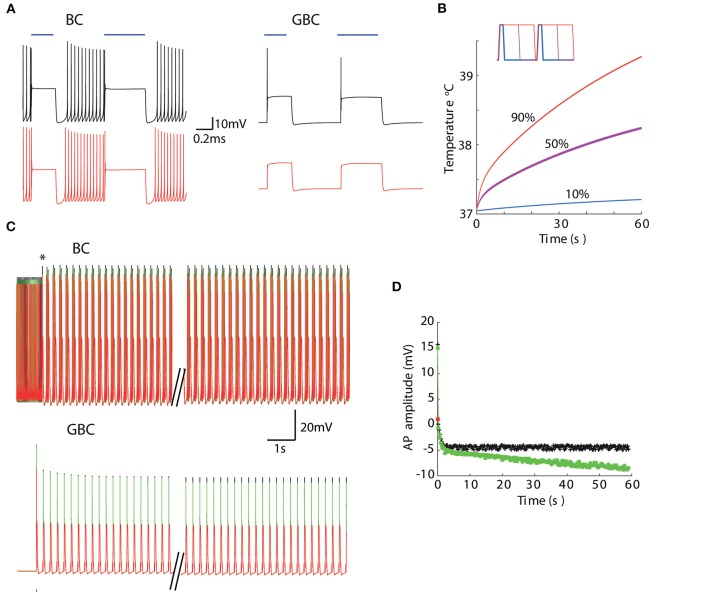
Temperature changes caused by light absorption affects membrane response to photocurrents. **(A)** Membrane potential of a basket cell (BC) and a dorsal cochlear nucleus bushy cell (GBC) models to 10 *mW*-473 *nm* light pulses at 37°C (top) and 39°C (bottom). **(B)** Temperature at 10 μ*m*for 4 Hz stimulation (20 *mW*) for 10% (blue), 50% (magenta) and 90% (red) duty cycles (inset shows 0.5 s pulses with the three different duty cycles). **(C)** BC and GBC responses for 10% duty cycle (4 *Hz*) light pulses with fixed temperatures (37°C black and 39°C red) and when temperature raises (green) in response to light pulses (black trace in **B**). **(D)** Action potential amplitude evolution in time of GBC model in response to light pulses in **(C)**. The red square is the amplitude of the single AP the GBC model fired when temperature was set to 39°C.

We then combine temperature and irradiation in modeled neurons that also contained a channelrhodopsin-2-driven photocurrents (Wang and Buzsáki, 1996; Williams et al., 2013). We have used two distinct cell models to illustrate the interaction of channelrhodopsin photocurrents with other ionic currents in the neuron. The basket cell shows high-frequency firing that increases proportionally to the injected current (Martina et al., 1998) and a bushy cell of the dorsal cochlear nucleus that show single action potentials in response to continuously injected currents (Leao et al., 2006). At 1 *mW* power, the basket cell model fired action potentials at the beginning of each pulse whether at 37°C or 39°C (Figure 6A). However, the bushy cell model only fired APs at physiological temperature (Figure 6A). The tissue reaches 39°C quickly for duty 50% or 90% duty cycles, but the temperature only rises mildly for 10% duty cycle (Figure 6B). Nevertheless, even at 10% duty cycle, bushy cell light-elicited AP amplitude is still affected by the small increase in temperature (Figures 6C,D). Taken together, this data suggests that temperature can alter the efficiency of photocurrents in eliciting APs. Most importantly, the effect of temperature and light stimulation interaction in the membrane is greatly dependent on native voltage-gated channels.

## Discussion

In the context of optogenetics, the first study that addressed the interaction of light emanating from an optical fiber with brain tissue omitted absorption (Aravanis et al., 2007). Aravanis and colleagues argued that the effect of light (400–900 *nm*) absorption could be neglected when simulating light transmission in the brain (Aravanis et al., 2007). However, while absorption does not affect significantly the spatial computation of light intensity (as most of the loss occurs through scattering), it is through absorption that heat is generated. Also, we opt to use the simpler Kubelka-Munk model for light transmission instead of a more accurate Monte Carlo method as the former generates values that approximate empirical results for short distances (~ 1 *mm*) (Aravanis et al., 2007; Džimbeg-Malčić et al., 2011).

Our bio-heat transfer results corroborate with recent studies found in the literature (Stujenske et al., 2015; Arias-Gil et al., 2016). These authors were the first to explores heat generation by light in optogenetic experiments and compare simulations with empirical measurements. Our work, instead, explore the effect of bio-heat transfer in neurons and networks, in particular, with a few differences compared to the study by Stujenske and colleagues (Stujenske et al., 2015). For instance, these authors used light absorption and scattering coefficients obtained from human brain tissue interpolated from different wavelengths while here we employ coefficients obtained from rodent brains in specific wavelengths used in optogenetic experiments (Bernstein et al., 2008; Stujenske et al., 2015). Besides, we have calculated temporo-spatial photon flux in brain tissue. Ultimately, photon flux determines the opening of channelrhodopsin pores, and these values could be directly used for simulation of channelrhodopsin activation (Zhang et al., 2015).

We used homogeneous absorption coefficients for a given wavelength, but it is clear from optical measurements that light is unevenly absorbed in the brain (Jacques, 2013). Thus, the temperature can also increase unevenly based on anisotropic absorption coefficients. Besides, blood vessels are not homogeneously distributed in all brain regions; therefore, spatial differences in temperature buffering will further complicate the network effect of heat generation by optical stimulation. In other words, the effect of the increase in temperature in optogenetic experiments will depend on the region, neuron type, and connections and can significantly affect neuronal processing. Minimizing stimulation time may help to prevent unwanted heat effects in neuronal function. In experiments where long stimulation times are desirable, step-function opsins may be the tool of choice for avoiding heat-related changes in firing and behavior.

The temperature effect in the gating of voltage-dependent channels is classically modeled by using an empirical factor (*Q*_10_) to multiply rate constants (incorporating temperature dependence to the classical Hodgkin and Huxley formalism) (Thompson et al., 1985). In addition, ion reversal potentials in semipermeable membranes are directly proportional to temperature. We simulated the effect of a 2°C change in a classical model of interneuron network gamma (ING) oscillation (Wang and Buzsáki, 1996). The idea that gamma oscillation arises from the interaction of fast spiking interneurons originated from slice and modeling studies (Whittington et al., 1995; Wang and Buzsáki, 1996) and it was demonstrated by a highly influential optogenetics study (Cardin et al., 2009). Cardin and colleagues elicited gamma oscillation in the neocortex by rhythmical optical stimulation of cells expressing the enzyme Cre recombinase (and channelrhodopsin) in a Parvalbumin-Cre animal (Cardin et al., 2009). To generate gamma oscillations, the authors optically stimulated neurons at the same frequency as the recorded local field potential (Cardin et al., 2009). It is known that rhythmical stimulation is likely to interfere with the local field potential recording due to the optoelectric effect (Mikulovic et al., 2016). However, the effect of temperature caused by optical stimulation in network responses is largely unexplored. Parvalbumin is especially found in soma targeting fast spiking interneurons (but it is also found in several other types of interneurons) (Klausberger et al., 2005; Mikulovic et al., 2016). Using Wang and Buzsaki's model of ING (1996), we found that an increase of two degrees significantly organizes the inhibitory neuron network. At 39°C, firing in gamma can be observed in less than 50 ms from the simulation onset (when firing of individual neurons is random) while at 37°C, that network takes almost 5 times longer to organize its spikes at gamma frequency. Also, network firing frequency increases in several *Hz*. Changes in gamma oscillation frequency by temperature has been observed experimentally (Leao et al., 2009), and as the increase in temperature depends on the proximity of targeted neurons to the optical fiber, light stimulation could generate small networks that oscillate incoherently from non-heated networks and this effect is not directly associated to opsin expression.

Here, we show that different types of neurons can have very different responses to similar light pulses. There has been little concern in optogenetic experiments regarding native currents of neuronal populations of interest (Adamantidis et al., 2015). However, we show that native voltage-gated currents can have a huge impact on how neurons fire to light stimulation. For example, neurons that express strong low threshold *K*+ currents to avoid repetitive firing when currents are injected will only fire one to a couple of spikes independent of the duration of the light pulse (Leao et al., 2008). On the other hand, fast spike neurons expressing high-threshold *K*+ currents like basket cells (Martina et al., 1998) will respond, most likely, with multiple spikes after each light pulse. Neurons with strong inward currents activated by hyperpolarization (e.g., Ih) could also produce strong depolarizations (and action potentials) by activation of Ih rather than the reversal of *Cl*− gradients (Leao et al., 2011; Adamantidis et al., 2015). It is important to note that the simple ChR2 model used here describes well the behavior of macroscopic photocurrents for short periods (that cover a large number of optogenetic experiments) (Williams et al., 2013). Hence, this ChR2 model could be added to specific cell models that are readily available in databases like the ModelDB (McDougal et al., 2017) for optimization of light protocol design.

Finally, temperature affects the transfer function of a given neuron according to the diversity of ion channels in it (Cao and Oertel, 2005). For that reason, while some neuron types increase spontaneous firing, other populations may become quiet when the temperature is changed (Kim and Connors, 2012). Most importantly, changes in temperature and native channels may hinder optogenetic stimulation. Our optogenetic simulations using the bushy cell model showed that light pulses are unable to elicit spikes when the cell is heated to 39°C. Bushy cells are known to express low threshold potassium channels (Kv1) (Rothman and Manis, 2003), and these channels prevent the firing of multiple APs in response to tonic currents (Couchman et al., 2011). Thus, accelerating the opening of Kv1 channels could prevent spike generation by photocurrents. However, the interaction of channelrhodopsin photocurrents with native voltage-gated currents of a given cell is a subject largely explored, especially when changes in temperature caused by the light stimulation affects the gating dynamics of native channels. Future studies should assess the interaction of photocurrents with native voltage-gated currents and examine the effect of temperature.

## Conclusion

In this work, we have used the finite element method to address brain temperature changes caused by light stimulation in optogenetics and its effect in neuron firing. We found that temperature can increase by about 2.6°C in 1 min for blue light stimulation (20 mW of power, Figure 3C). A two-degree change in temperature, when applied to a model of a spontaneous firing neuron, caused a dramatic increase in firing frequency and change in action potential shape. Conversely, a 2°C-increase in temperature in a fast spiking interneuron network model of gamma oscillation produced a large increase in neuronal synchrony and oscillation frequency. Moreover, the effect of channelrhodopsin-driven photocurrents on membrane potential is dramatically affected by temperature changes provoked by light stimulation itself, especially in the single-firing cell model.

In summary, we have shown that temperature increase caused by brain optical stimulation, with light intensities commonly used in optogenetic experiments (Cardin et al., 2009; Adamantidis et al., 2011) can considerably affect neuron and network properties independently of opsin expression. Moreover, the temperature can alter cellular responses to optical stimulation. As the usage of channelrhodopsin becomes widespread, studies tend to assume that optical stimulation elicits spiking activity without assessing cellular responses (Ahlbeck et al., 2018; Almada et al., 2018). Thus, the whole cell current- and voltage-clamp assessment of the cell response to optical stimulation may still be necessary to determine optimal light stimulation protocols.

## Data Availability Statement

The datasets generated for this study are available on request to the corresponding author.

## Author Contributions

HP performed the COMSOL/MATLAB modeling and simulations about light propagation in brain tissue. RC performed the optical theoretical analysis. TM modeled the interaction of channelrhodopsin photocurrents with the ionic currents in the neuron. RL modeled the synchrony in a gamma oscillation network during temperature changes. HP, RC, TM, and RL wrote the paper. The authors read and approved the final manuscript.

### Conflict of Interest

The authors declare that the research was conducted in the absence of any commercial or financial relationships that could be construed as a potential conflict of interest.
